# Early detection of lung cancer using artificial intelligence-enhanced optical nanosensing of chromatin alterations in field carcinogenesis

**DOI:** 10.1038/s41598-023-40550-6

**Published:** 2023-08-22

**Authors:** Ali Daneshkhah, Sravya Prabhala, Parvathi Viswanathan, Hariharan Subramanian, Jianan Lin, Andrew S Chang, Ankit Bharat, Hemant Kumar Roy, Vadim Backman

**Affiliations:** 1 Department of Biomedical Engineering, Northwestern University, Evanston, IL USA; 2 NanoCytomics, Evanston, IL USA; 3 Baylor College of Medicine, Houston, TX USA; 4 Department of Surgery, Feinberg School of Medicine, Canning Thoracic Institute, Northwestern University, 420 East Superior Street, Chicago, IL 60611 USA

**Keywords:** Biophysics, Biotechnology, Cancer, Structural biology, Biomarkers, Diseases, Health care, Medical research, Risk factors, Optics and photonics

## Abstract

Supranucleosomal chromatin structure, including chromatin domain conformation, is involved in the regulation of gene expression and its dysregulation has been associated with carcinogenesis. Prior studies have shown that cells in the buccal mucosa carry a molecular signature of lung cancer among the cigarette-smoking population, the phenomenon known as field carcinogenesis or field of injury. Thus, we hypothesized that chromatin structural changes in buccal mucosa can be predictive of lung cancer. However, the small size of the chromatin chain (approximately 20 nm) folded into chromatin packing domains, themselves typically below 300 nm in diameter, preclude the detection of alterations in intradomain chromatin conformation using diffraction-limited optical microscopy. In this study, we developed an optical spectroscopic statistical nanosensing technique to detect chromatin packing domain changes in buccal mucosa as a lung cancer biomarker: chromatin-sensitive partial wave spectroscopic microscopy (csPWS). Artificial intelligence (AI) was applied to csPWS measurements of chromatin alterations to enhance diagnostic performance. Our AI-enhanced buccal csPWS nanocytology of 179 patients at two clinical sites distinguished Stage-I lung cancer versus cancer-free controls with an area under the ROC curve (AUC) of 0.92 ± 0.06 for Site 1 (in-state location) and 0.82 ± 0.11 for Site 2 (out-of-state location).

## Introduction

Cancer screening tests should, ideally, identify cancer before symptoms have appeared and while the tumor is small in order to effectively increase the chance of treatment and reduce mortality. Lung cancer is the leading cause of cancer deaths across races and genders in the U.S. with an overall 5-year survival rate of 22.9% which is notably lower than colorectal (65.1%), breast (90.6%), and prostate cancers (96.8%)^1^. However, if lung cancer is detected at an early stage it is highly curable through surgical resection. The 5-year survival rate for late-stage (distant) non-small lung cancer (NSLC) is less than 8% but improves to 64% if detected at a localized stage, and reaches 80% if detected at Stage-IA^2^. Low-dose computed tomography (LDCT) has been established as the gold standard for lung cancer screening and is associated with a 20% decrease in mortality among patients screened with the technique. Accessibility, cost, stigma, and lack of adherence to LDCT guidelines are among the major challenges limiting its impact, as only about 5% of the LDCT-eligible population undergoes screening^3^, resulting in 55% of lung cancer cases being detected at an advanced stage where the survival rate is below 8%^4^. We therefore propose a minimally invasive, accessible, sensitive, and accurate screening test with high sensitivity (Se) to early-stage lung cancer.

Screening methods other than LDCT such as chest X-rays and sputum cytology have proven unsatisfactory when evaluated in large-scale clinical screening settings^5^. New methods based on standard protein biomarkers used for the detection of cancer do not provide sufficient sensitivity and specificity (Sp)^6^. Recently, there has been significant interest in the development of protocols that rely on tumor secretions in the blood, such as liquid biopsy. Tests being developed by companies including Grail, Freenome, Guardant, Delfi, and Thrive identify cancer by analyzing circulating tumor DNA (ctDNA) or tumor-derived circulating free DNA (cfDNA) properties such as gene mutations, methylation, and fragmentation^7–11^. Although initial results have shown promise in the detection of various cancers, including lung cancer, the sensitivity to Stage-I and smaller lesions drops precipitously below a clinically acceptable level. It has been suggested that this is not primarily a technological limitation, but may instead be related to the biology of the source and type of biomarker. Smaller lesions secrete less tumor ctDNA (~ 1 ctDNA/ 10 mL of blood), while tumor heterogeneity can only be modeled through many tumor-byproduct biomarkers, which makes it challenging to find the needed quantities of ctDNA in a clinically practical blood sample^12^. For example, the overall sensitivity of the Grail multi-cancer early detection (MCED) test drops from 90.1% [95% confidence interval (CI) 87.5–92.2%)] in Stage-IV patients to 16.8% [95% CI 14.5–19.5%] in Stage-I patients^13^. Liquid biopsy can be a powerful tool for non-screenable cancers (pancreatic, etc.) but for cancers with established screening protocols, such as colorectal and lung, methods to detect highly treatable early-stage lesions are still urgently needed. To address these issues and develop an effective screening test for lung cancer, we optimized three crucial aspects: (1) biomarker source, (2) biomarker type, and (3) enabling technology.

An ideal biomarker source for the development of a large-scale screening test should be obtainable through a minimally invasive procedure, with an easy-to-implement and reproducible protocol, and provide high sensitivity to small treatable lesions^14^. Our approach to finding this biomarker source relies upon the application of a well-established phenomenon known as field carcinogenesis (or field effect, field of injury) which was first introduced six decades ago^15^. In field carcinogenesis, the genetic/epigenetic alterations leading to neoplastic cell transformation are distributed diffusely throughout the “field of injury” even at the pre-malignant stage^15–23^. In molecular field carcinogenesis, tumors arise on a histologically normal-appearing, phenotypically silent, but preconditioned and premalignant ‘field’. This field carries transcriptomic, genomic, and epigenetic alterations, which can be indicative of an ensuing neoplasm within the affected region^20,24^.Due to the stochastic nature of these molecular changes, some cells may eventually give rise to a tumor clone. Thus, in lung field carcinogenesis, cells throughout the entire aero-digestive mucosa harbor molecular biomarkers of carcinogenesis regardless of their proximity to a tumor^16,17^. The buccal mucosa is widely recognized as a “molecular mirror” for lung cancer because of field carcinogenesis^16,18,19,25^ and we considered it as our biomarker source for two reasons. First, buccal (cheek) brushings are easily performed and uniquely suited for an at-home test or for a primary care office, dentist, etc., as opposed to “liquid biopsies” that can hardly be self-administered. Next, due to the etiological relationship between field carcinogenesis and the rise of tumors on this molecular background, as a biomarker field carcinogenesis is expected to be highly sensitive to early (e.g., Stage-I) cancers, regardless of tumor size, which is diagnostically crucial and an important difference from other sources such as blood or breath which depend on the load of secretions by a tumor, and thus are more sensitive to large tumors than small ones.

Determining a suitable lung cancer biomarker type from buccal mucosa is the next major challenge. Biomarkers obtained from genetic changes are negatively impacted by the extremely high number of genetic alterations and astonishing tumor heterogeneity that hampers the application of downstream biomarkers for detection of small lesions. On the other hand, dynamic chromatin structure is a regulator of global patterns of gene expression, affecting the binding constants of transcriptional reactants, their diffusion to the sites of transcription, and gene accessibility to the reactants, including transcription factors (TF) and RNA polymerases (RNAPs)^26,27^. In particular, chromatin structure has been shown to be a regulator of cellular transcriptional plasticity, which is one of the critical etiological hallmarks of carcinogenesis, making it a potential candidate biomarker for early-stage lung cancer detection^26–28^.

To understand what types of chromatin structure may foster carcinogenesis, we first needed to calculate a quantifiable metric of chromatin structure. We and others have reported that chromatin is organized as a variety of packing domains^29–31^. At the smallest length scale, DNA wraps around histones and forms ~ 11 nm nucleosome complexes of “beads on a string” which are further folded into the curvilinear chromatin chain, between 5 and 24 nm^32^. These chromatin chains are packed together in various structural compactions and densities forming irregular blocks of larger packing domains. The packing domains have heterogeneous morphological properties with an average radius of 80 nm and genomic size of about 200 kbp^33^. Within these domains, chromatin shows a polymeric fractal-like behavior (i.e., the mass scaling behavior within domains follows a near-power-law relationship) along with radially decreasing mass density from the center to the periphery^33^. Chromatin packing scaling (D) is defined by estimating the number of base pairs ($N$) scaling with the radius of the occupied volume ($R$) as $N\alpha {R}^{\mathrm{D}}$. Experimentally measured values of D fall between 5/3 and 3 across packing domains^33^. A higher D value may indicate a packing domain with an increased chromatin heterogeneity and a decreased gene connectivity, resulting in more frequent longer-distance contacts^34,35^. Chromatin domain structures with a higher D have been linked to further upregulation of initially upregulated genes and concomitant suppression of downregulated genes^26,34^. In turn, these processes result in transcriptional patterns with greater transcriptional malleability and intercellular transcriptional heterogeneity^26,33^. As neoplastic cells must keep developing new traits in response to stressors (e.g., hypoxia, immune system attack, new microenvironment, chemotherapy), they benefit from transcriptional plasticity. Tumor cells that can more efficiently upregulate critical pro-survival pathways for a given level of stress through transcriptional malleability and heterogeneity have a higher likelihood of attaining a rare transcriptional state that is critical for cancer cell survival, thus further carrying this transcriptional phenotype through replication and increasing the probability of their progeny to acquire other gene mutations, some of which may be beneficial to tumorigenesis. Thus, transcriptional plasticity-facilitating chromatin states (including higher chromatin packing domain $\mathrm{D}$) may play a critical role in creating a “proneoplastic positive feedback loop” and therefore serve as a marker for neoplastic progression^35^. A significant correlation between proneoplastic processes with higher packing scaling D, as well as transcriptional plasticity across different malignancies, supports the concept of chromatin-regulated transcriptional plasticity. In particular, a comprehensive analysis of the TCGS (The Cancer Genome Atlas) database revealed that transcriptional divergence in late-stage (Stage-III–IV) tumors at the time of diagnosis is an independent predictor of survival time among patients with lung, colon, and breast cancer^26^.

Chromatin structural changes occur from across the chromatin chain to domains at length scales from ~ 20 to ~ 300 nm, which is too small to be observed by conventional optical microscopy. In order to reproducibly measure these sub-diffractional chromatin alterations, we developed a new technique called chromatin-sensitive partial wave spectroscopic (PWS) microscopy, based on the physical principles of statistical spectroscopic nanosensing. csPWS is a fast, reliable, and nanoscale-sensitive optical spectroscopic technique that can detect chromatin conformation changes with sensitivity between 23 and 334 nm^36^. The key innovation in csPWS is statistical nanosensing in which sub-diffractional structures, while not resolvable through conventional optical microscopy, are detectable through analysis of the spatial variations of the refractive index (RI) via the spectroscopy of scattered light interference within each of the microscopic resolution voxels^25,37–42^. The output of csPWS microscopy is an image of the cell nucleus where the spectrum resulting from the interference of light scattered by the subdiffractional spatial variations of chromatin density with a reference wave is processed to measure chromatin packing scaling D^30,33,43^.

D describes a quantitative statistical measurement of the three-dimensional packing of the chromatin polymer within a self-similar domain. However, local physical conditions such as nuclear crowding density, genomic size (*N*_d_), domain volume fraction, and domain intracellular positioning (peripheral vs. interior, etc.) are also important physical regulators that help determine chromatin connectivity, accessibility, and transcriptional plasticity, and therefore, gene expression^26,44^. As packing scaling D is not the only predictor of plasticity-fostering conformation, calculating average D will not fully capture the complexity of the chromatin regulatory mechanisms influencing gene expression. Thus, we utilized advanced machine learning algorithms and artificial intelligence (AI) to distinguish the biological footprints of lung cancer contained in the images of nuclear D. Such a novel “hybrid” AI + etiological biomarker approach is made possible—and potent—by developing neural network (NN) layers informed with mechanistic data obtained from the chromatin structure alterations contained in the packing scaling D image. In this fashion we coupled our novel csPWS microscopy with a knowledge-based AI approach and achieved high sensitivity for the detection of early-stage lung cancer.

## Material and methods

csPWS nanocytology involves the collection, shipment, and preparation of buccal samples followed by csPWS image acquisition and evaluation of the nuclear chromatin packing scaling D image using AI enhancement.

### Patient recruitment

Patients were recruited through Institutional Review Boards approved at Northwestern University, Northwestern Memorial Hospital, and Boston Medical Center/Boston University. All methods were performed in accordance with the relevant guidelines and regulations and written informed consent was obtained from all participants. The cohort comprised 96 patients with histologically confirmed lung cancer within 1 year prior to recruitment (case population) and 83 patients with a negative LDCT scan within 1 year prior to recruitment (control populations). 167 patients were over 45 years of age, nine patients were 27 to 44 years of age, and the age of three patients was unknown. Exclusion criteria were family history of lung cancer, exposure to chemotherapy and radiation in the past 3 months, pregnant/lactating women, and inability to give informed consent. Our data were obtained with discovery and independent validation of datasets from Site 1, Northwestern Memorial Hospital (NMH) in Chicago, Illinois, US, and Site 2, Boston Medical Center (BMC) in Boston, Massachusetts, US. The control population included non-smokers, low-risk and high-risk smokers, and patients with benign nodules. The lung cancer patients included all stages but were predominantly Stage-I patients (62% for Site 1 including 11% Stage-IA, and 76% for Site 2, including 14% Stage-IA).

### Sample collection

Buccal samples were collected in the primary care physician’s office through a buccal swab procedure using a minimally invasive standard of care (Cytobrush, CooperSurgical, Inc., Trumbull, CT, USA). The patients rinsed their mouths with water three times before the physician placed the bristles on the inside of one buccal surface followed by a top to down motion including brush rotation. Next, the impregnated swabs were dipped into 1.5 ml vial tubes (Neptune Scientific, San Diego, USA) containing 750 ml of 25% ethanol (collection buffer). The samples were then packaged and shipped to the central lab for csPWS microscopy.

### Sample shipment

The Site 2 samples were shipped through the air from an out-of-state location while the Site 1 samples were shipped by ground transportation from an in-state location. The samples were maintained at a temperature below 10 °C during transport using a custom-built transport kit and were received at the central facility within 24 h of sample collection. The transportation kit included an outer corrugated box (Uline, Pleasant Prairie, WI, USA) and polar pack refrigerants (SONOCO Thermosafe, Arlington Heights, IL, USA) and temperature was monitored using a temperature indicator (Timestrip, Cambridge, UK). The sealed vial was packaged using an inner Styrofoam container and absorbent sheets to avoid possible leakage under refrigerated conditions.

### Sample preparation

Clinical samples were prepared within 24 h of collection based on the approaches reported earlier^45^. In brief, the samples in 25% ethanol were spray deposited on a Fisher brand Superfrost microscope slide (Fisher Scientific, Hampton, NH, USA) using our custom-built cell deposition system to form a non-overlapping monolayer of buccal cells. The sample slide was air-dried prior to cytological fixation with 95% ethanol (Thermo Fisher Scientific, Waltham, MA, USA) followed by csPWS microscopy.

### Standard operating procedure (SOP)

**Figure 1:**
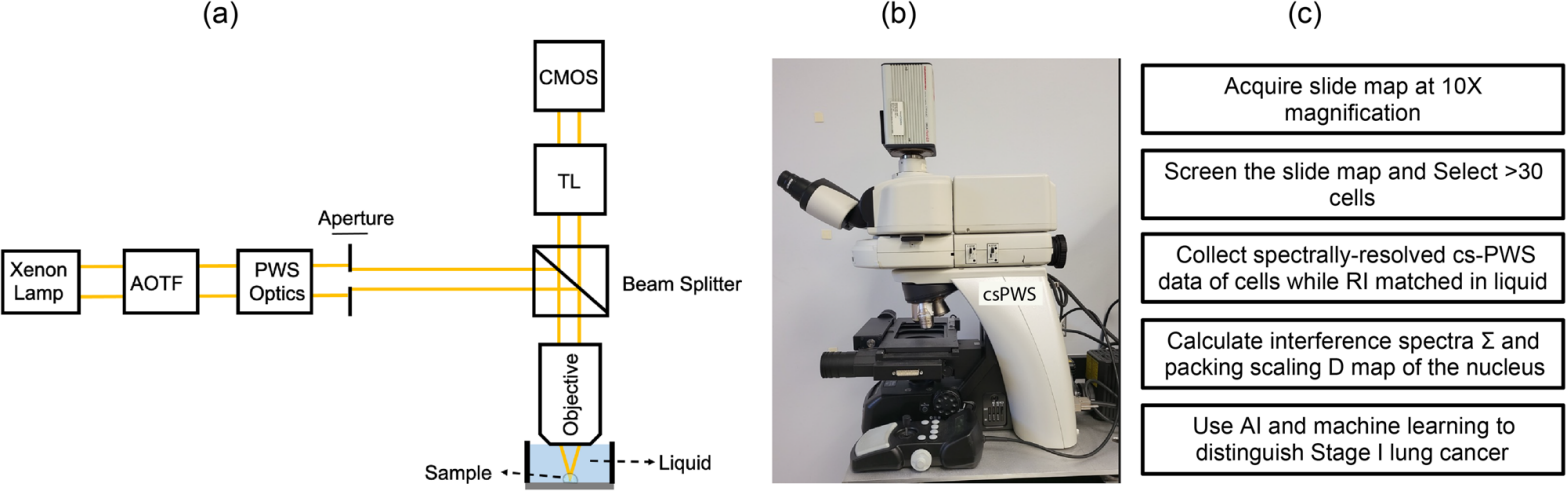
(**a**) Schematic of csPWS instrument. Tube lens (TL), Acousto-optic tunable filter (AOTF), Complementary metal oxide semiconductor (CMOS).  (**b**) csPWS instrument (**c**) workflow of csPWS buccal nanocytology.

We developed a csPWS SOP to capture buccal nuclear chromatin structural changes. Our goal was to ensure a fast, robust, reliable, and repeatable protocol with small variability of physical features of the cells acquired by csPWS from each patient. To minimize the complexity at the collection site, we carried out the cell fixation and sample deposition at the central lab instead of the primary care office^45^. For each patient, a total of > 30 cells were collected, where the sample size number was determined by power analysis with the confidence interval (CI) on mean D restricted to less than 5% of the difference between cancer and control population^45^. We created a sample transport solution of 25% ethanol and used our custom-built cell deposition device to spray deposit a non-deformed, non-overlapping monolayer of buccal cells with clear nuclear boundaries on the glass slide. An airdrying step enhanced the attachment of cells to the glass, followed by fixation with 95% ethanol and csPWS microscopy. The csPWS microscope was controlled via custom software with a graphical user interface (GUI). The imaging procedure began by scanning the whole slide using a 10X air objective. A semi-automated slide-map module was developed to rapidly generate a low-magnification image by collecting and stitching individual slide region images. This assisted a trained user blinded to the diagnostic information in selecting over 30 buccal cells across the entire slide in a timely manner. Our cell screening protocol selected non-folded and non-overlapping cells with clear nucleus boundaries. The csPWS spectral acquisition was performed with the cells in a liquid medium (95% ethanol) using a liquid-dipping 40X optical objective (Nikon, Melville, NY, USA) to match the RI between the buccal cell and liquid cover (shown in Fig. 1a). The csPWS acquisition algorithm automatically acquired spectral data for selected cells, and the analysis algorithm rapidly generated the processed spectral data. These processes facilitated reliable and reproducible results, making csPWS suitable for larger future studies that include additional clinical sites.

### csPWS instrument

The design and schematic of the csPWS instrument and the optical path for collecting buccal cell data are shown in Fig. 1a. The csPWS optical system (shown in Fig. 1b) is built on a commercial microscope (Nikon Instruments, Melville, NY, USA) using a Nikon Eclipse Ni-E microscope body with modifications made to include a Xenon lamp (Exceliatas, Tampa, FL, USA). The light is fed to an acousto-optic tunable filter with a switching speed of 50 μs, a bandwidth of 3 nm, and a spectral range of 450–700 nm (Gooch and Housego, UK). The light passes through objective lenses (Nikon, Melville, NY, USA) that are attached to an automated objective turret and onto a sample that is positioned with a nanomotion piezo-stage (Prior Scientific, Rockland, MA, USA). The data is recorded through a digital CMOS camera, ORCA Flash 2.8 (Hamamatsu, Bridgewater, NJ, USA) thereby enabling hyperspectral imaging. High-throughput and automated csPWS acquisition are obtained by utilizing Kohler illumination for uniform sample illumination. We use a high-speed, high-resolution RGB camera (Thorlabs Inc., Newton, NJ, USA) for low-magnification slide mapping (UPlanFL N 10x, Olympus). The integration of a high-speed camera with an advanced sample stage significantly expedited the csPWS data collection procedure. We further enhanced efficacy by developing a custom-built software (MATLAB, Mathworks, Inc). This software performs a rapid slide mapping in low magnification (UPlanFL N 10x, Olympus), followed by precise autofocus on individual cells and automatic collection of spectrally resolved data from the entire nucleus of over 30 cells in a timely manner. The workflow of csPWS nanocytology is presented in Fig. 1c. A significant advancement of csPWS lies in the utilization of mathematical modeling for estimation of chromatin packing domain conformation, which was confirmed through simulation and experimental results^43^.

### csPWS microscopy

Conventional microscopy systems are unable to resolve structures smaller than 200 nm (half the wavelength of light). Our lab developed csPWS, an optical statistical spectroscopic nanosensing approach for the detection of chromatin packing domain changes in the nucleus of buccal mucosa to distinguish histologically normal buccal cells that may carry a signature of cancer. csPWS is configured so that a spatially-varying RI material, such as the nucleus of buccal cells, is sandwiched between two semi-finite homogenous media of glass and 95% ethanol. csPWS acquires a high magnification of monochromatic spectrally resolved images between wavelengths of 450–700 nm and distinguishes non-resolvable sub-diffractional length scales through the spectroscopic analysis of scattered light. For a given location ***r*** within a cell, the local RI is proportional to the local macromolecular density (ρ) of proteins, DNA, and RNA as shown in Eq. (1)^45,46^.

1 $$n({\mathbf{r}}) \, = n_{media} + \alpha \rho ({\mathbf{r}})$$

where α is the RI increment, which is nearly independent of macromolecular chemical composition within the accuracy of measurements. csPWS uses liquid cover microscopy to nearly match the RI between the buccal cell and liquid cover (shown in Fig. 1a) while creating a mismatch between the cell-glass interface. Thus, the scattering of light from a reference wave is influenced by the nanoscale heterogeneity and density variation of the intracellular macromolecules. Matching the RI between the buccal cell and liquid cover minimizes the contribution of cell surface roughness on the csPWS signal while maximizing the contribution of the signal arising from intercellular structural changes.

To quantify the 3D RI fluctuation of the weakly scattering buccal nucleus, csPWS calculates the standard deviation of the interference spectra (Σ) resulting from interference of the reference wave reflected from the cell-glass interface and light scattered from all spatial variations of the RI due to nanoscale variations of chromatin density within the coherence volume formed by diffraction in the transverse plane (458 × 458 nm^2^, Abbe formula) and the depth of field^47^ (2874 nm) longitudinally^33,43^. Σ is proportional to the Fourier transform of the autocorrelation function (ACF) of ρ(***r***) integrated over the Fourier transform of the coherence volume. Each csPWS image stack is normalized by the reference wave that is acquired at the interface of the glass and cover media from a blank region on the slide. Given the instrumentation parameters related to light illumination and collection geometry, we estimated the packing scaling D value from Σ using the analytical framework for quantifying chromatin structure with spectral microscopy provided in^43^. The Σ signal measured by csPWS is proportional to the mass density distribution of chromatin (B(r), where r is the spatial separation) convolved with a smoothing function S(r), which is characterized by the optical system setup. Assessment of chromatin transmission electron microscopy with ChromEM labeling (ChromTEM) data in lung adenocarcinoma A549 cells and differentiated BJ cells led us to model chromatin mass density distribution by a modified power-law autocorrelation function controlled by model parameter D_b_^43^. Born approximation was employed to describe the smoothing function S(r) based on microscope numerical aperture, source spectrum and cell sample characteristics such as light illumination/collection numerical aperture, depth of field and cell thickness, forward and reverse Fresnel transmission and reflection coefficients at the cell/glass interface, RI of media, density of chromatin and macromolecular crowding, CVC, and genomic length^43^. Employing the Laplace transform within the fractal regime, we were able to obtain model parameter D_b_ for a given sigma value which led us to the calculation of mass density ACF. Packing scaling D was calculated from the derivative of the mass density ACF function based on Eq. (2)^43^.

2 $$\mathrm{D }= 3 +\frac{\partial (Log (B\left(r\right)))}{\partial (Log \left(r\right))}$$

The accuracy of this approach was confirmed through comparison with measurements of average D by ChromTEM, and a computational chromatin model using finite-difference time-domain (FDTD) simulations^43^. csPWS length scale sensitivity depends on illumination-collection geometry. We used a small-to-moderate numerical aperture (NA) of light incidence of 0.6 and light collection NA of 0.8 for csPWS. This illumination setting ensures a uniform intensity across the sample plane due to the Köhler alignment^48^ and delivers a chromatin length scale sensitivity of 23–334 nm (the exact value depends on the sample structure and thickness)^36,49^. The larger length scales have a negligible effect on csPWS output signal^45,50^. Electron microscopy data has revealed buccal chromatin structures significantly altered at this length scale range^51^. Thus, csPWS nanocytology is primarily sensitive to length scales that are not resolvable by conventional optical microscopy, but do carry a profound signature of field carcinogenesis.

### AI analysis of packing scaling D

**Figure 2:**
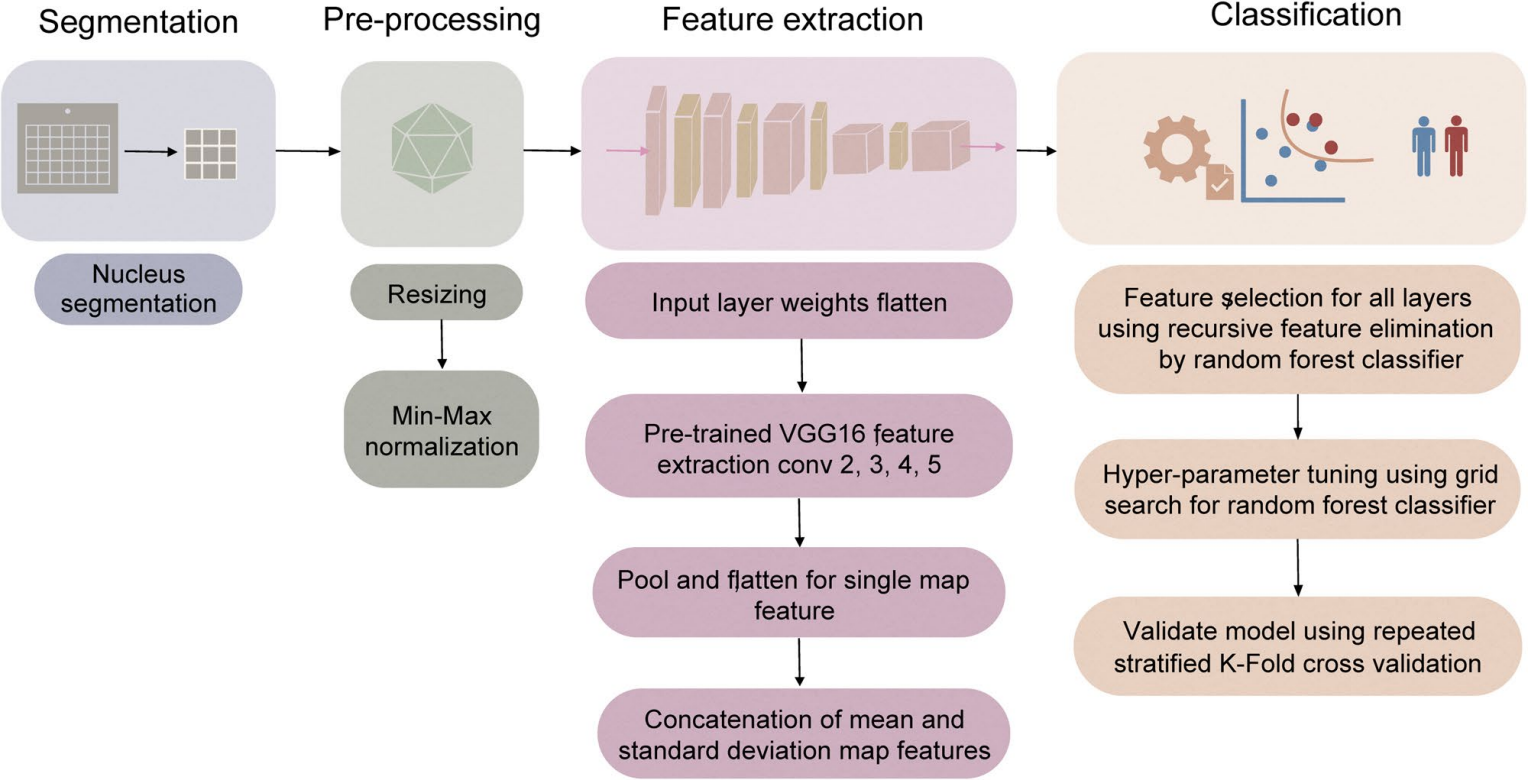
Workflow and architecture of the feature extraction and classification steps.

We used AI with csPWS data to determine whether it is possible to detect field carcinogenesis of buccal mucosa in patients with lung cancer and distinguish alterations in buccal chromatin packing domains that indicate tumor initiation and progression. Our AI-enhanced approach consisted of nuclear segmentation, preprocessing, feature learning, and classification of csPWS images as shown in Fig. 2. Nearly 7000 buccal csPWS D images (960 × 720 pixels) from 179 patients were evaluated in this study. Nuclear segmentation was conducted by a trained user blinded to the diagnostic information, and outlier cells with deformed shapes were excluded. The trained user relies on image features for detecting the region of interest, which is the whole nucleus, by evaluating contrast between the nuclear boundary and cytoplasm. Additionally, nuclei typically have characteristic shapes, such as round or elliptical, and distinctive textures such as that of cytoplasm, which further help the user to locate the intact boundary of the entire nucleus. Next, the nuclei images were resized and passed through min–max normalization in our preprocessing subsection. We utilized a pre-trained deep learning algorithm to extract characterizing features from the D-image while identifying the diagnostic features using a supervised feature selection method. For the feature extractor unit, we incorporated transfer learning on VGG16 architecture, a convolutional neural network (CNN) pre-trained on 14 million images belonging to 1000 different labels from the ImageNet data set. Out of the 13 Convolutional layers and five Max Pooling layers of the VGG16 model, we extracted features from the final convolutional layers in blocks 2 through 5. The mean and standard deviation of the flattened features across all cells belonging to a patient were calculated to create a feature vector. We used a multiple instance learning (MIL) method of instance-level feature aggregation that facilitated the usage of patient-level clinical ground truths. MIL enables a seamless process integration for pathologists^52^ and for that reason was used in our approach. To further reduce the dimension of the patient feature, we conducted a recursive feature elimination method using a random forest algorithm, thus selecting a panel of 40 features with enhanced classification properties. We used a parameter-tuned random forest classifier to determine patients with lung cancer from the control populations using a patient-wise analysis of the diagnostic features obtained by CNN. The tuning of the classifier model for optimal parameters was performed using grid search by searching through iterations of multiple configurations, of which the model configuration with minimal error for our dataset was used for classification. For a robust evaluation of model performance on our relatively small dataset, we calculated our metrics AUC, sensitivity, and specificity using a stratified fourfold cross-validation method with 5 iterations.

## Results

### csPWS buccal D for detection of lung cancer (Site 1)

**Figure 3:**
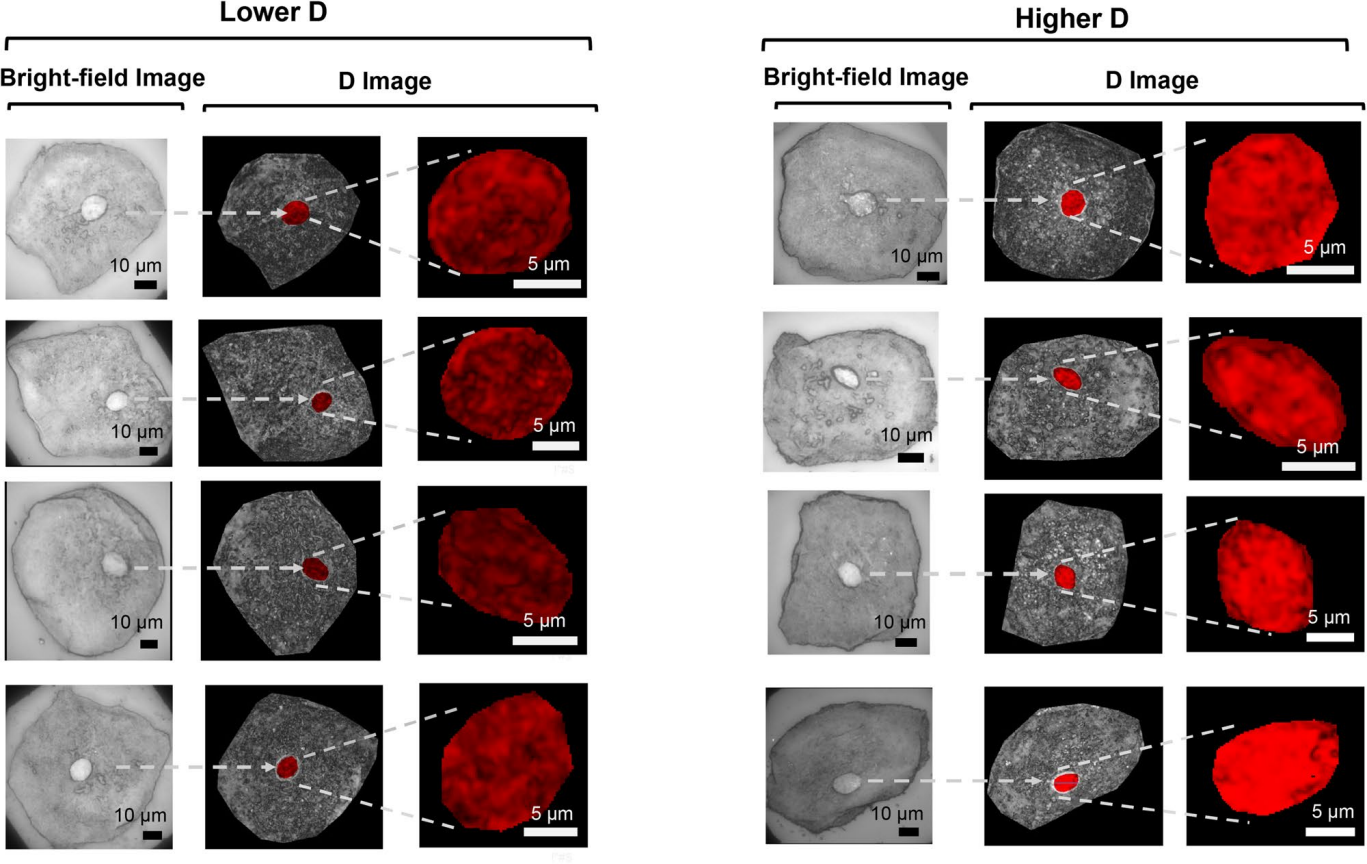
Brightfield image (first) the D image distribution (second and third) in eight cells from a control patient (left) with lower D and from a patient with lung cancer (right) with higher D. Nucleus D domains are highlighted in Red.

We first analyzed csPWS D images of clinical buccal samples in a double-blind case–control study from clinical Site 1. Most patients identified with lung cancer at this site were at Stage-I: 26 of the 42 (62%). The percentage of female patients with lung cancer was 56%, and 80% of subjects were Caucasian. We characterized average packing scaling D and evaluated the impact of demographic variables and smoking history, as well as association with lung cancer stage. csPWS D images of eight histologically normal buccal cells (confirmed by bright-field image) show intercellular domain variation and an overall increase in D in cells belonging to patients with lung cancer in comparison to a smoking control (Fig. 3).

**Figure 4:**
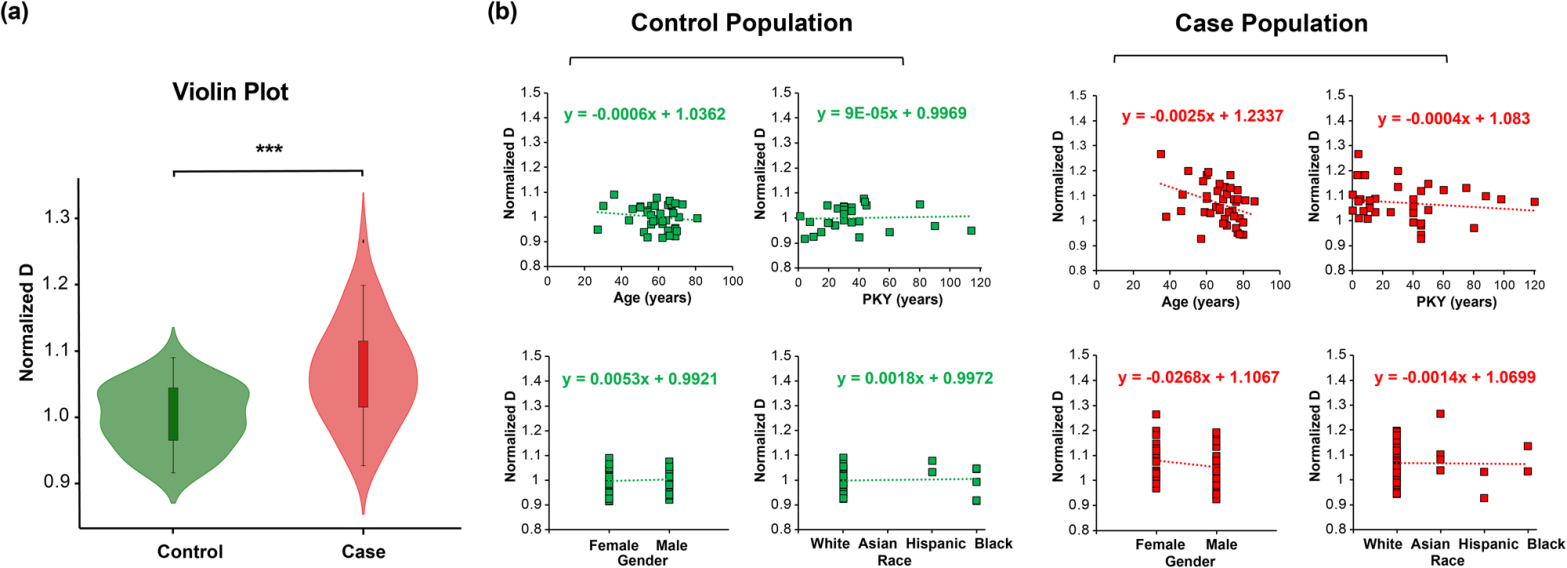
(**a**) Violin plots demonstrated the population range and distribution of normalized D in control (n = 40) and case (n = 42) population. (**b**) Linear regression analysis assessed the impact of demographic factors on average packing scaling D with the control population and case population.

The violin plot in Fig. 4a demonstrates the distribution of nucleus average D (normalized by control) and suggests an overall higher packing scaling D value for patients with lung cancer compared to the control population. Patient demographics including age, pack-years (PKY) of smoking, gender, and race, and their association with average D, were evaluated with the significance criterion of the p-value using ANCOVA (shown in Table 1**)**.

**Table 1:** Demographic factor distribution for Sites 1.

Demographic factor	Control parameter	Cancer parameter	*p*-Value
	Site 1	Site 1	Site 1
Age	59 ± 11	67 ± 12	0.01
Pack-years	35 ± 26	37 ± 30	0.52
Gender (% female)	49%	56%	0.47
Race (% Caucasian)	80%	80%	0.56

The average value and standard deviation are reported.

ANCOVA showed no statistically significant relationship between gender, race, and smoking pack-year with average packing scaling D, but age presented a slight negative correlation with a statistically significant relationship. We demonstrate scatter plots of patients with known demographic in Fig. 4. The slight slope of − 0.006 in the regression line for the control population shown in Fig. 4 indicated a minimal impact of advancing age on average D, where the change in D for aging 20 years is less than 18% of the difference in D between the case and control population. Applying an age adjustment confirmed a minimal influence on the diagnostic performance of average buccal D for lung cancer detection, with a negligible increase in the AUC from 0.76 to 0.77 (Supplementary material). Our regression lines shown in Fig. 4b demonstrated a negligible slope across genders in both control (0.005) and case population (-0.027). Similarly, trivial slopes of 0.002 and − 0.001 were identified across different races in control and case populations, respectively. This suggests gender and race do not alter the diagnostic ability. Pack-year smoking showed notably small slope of 9 × 10^–5^ and − 4 × 10^–4^ among the control and case population.

**Figure 5:**
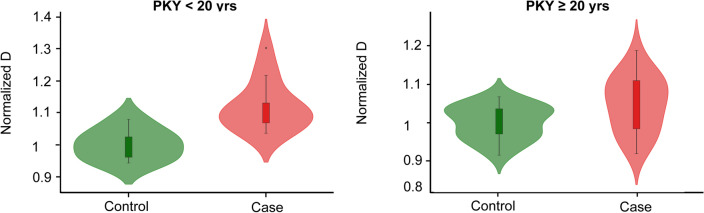
Population range and distribution of normalized D among patients with known smoking pack-year. Normalized D increased for patients with cancer (n = 14) in comparison with control population (n = 7) in low-risk smokers with PKY < 20 years (*p*-value = 0.001). Similarly, Normalized D increased for patients with cancer (n = 20) in comparison with control population (n = 20) in high-risk smokers with pack-year ≥ 20 years (*p*-value = 0.017).

We further evaluated the possible impact of intense and prolonged smoking on D. Figure 5 shows the distribution of buccal D among individuals with low smoking risk (pack-year < 20 years, typically ineligible for LDCT screening) and high smoking risk (pack-year ≥ 20 years, eligible for LDCT screening). Notably, patients with lung cancer demonstrated a higher D than the control population in both LDCT-eligible and ineligible populations, with the increase in D among lung cancer patients overshadowing the negligible effect of intense prolonged smoking. ($\Delta D$ for 20 pack-years < 20% ${\Delta D}_{cancer-control}$). This finding suggests that average buccal D is increased for patients with lung cancer in both low-intensity smoking LDCT-ineligible patients as well as high-intensity smoking LDCT-eligible patients, regardless smoking history.

### AI-enhanced csPWS for early-stage lung cancer detection

**Figure 6:**
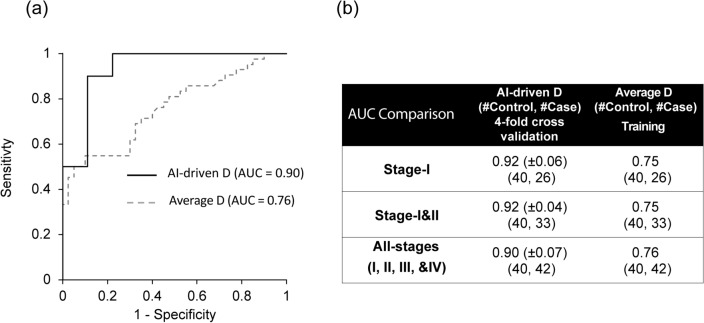
(**a**) ROC curve for AI-enhanced csPWS (in solid black) in comparison with average nucleus D in dashed grey (42 cases, 40 controls). (**b**) Comparison of the diagnostic performance assessed by AUC of AI-enhanced D in comparison with the nucleus average D for early-stage and all-stage lung cancer.

AI-enhanced csPWS nanocytology was developed to optimize packing domain data of nucleus buccal mucosa for detection of Stage-I lung cancer*.* We evaluated the performance of the AI-enhanced csPWS nanocytology method on Site 1 data and observed significant improvements in the diagnostic performance, as demonstrated by AUC when compared to the non-AI, nuclear average D protocol. The ROC curve of AI-enhanced D presented a notably higher AUC of 0.9 compared to an AUC of 0.76 obtained for average nuclear D (Fig. 6a).

A similar trend was observed for the detection of early-stage (Stage-I or Stage-I & II) lung cancer, where AI-enhanced csPWS nanocytology demonstrated a significantly higher AUC than the method based on the calculation of an average nuclear D (Fig. 6b).

### Validation of AI-enhanced csPWS nanocytology (Site 2)

We conducted an evaluation of AI-enhanced buccal D performance using samples acquired from Site 2, which was located out-of-state (BMC, Boston, MA). Patient demographics are shown in Table 2. Most patients identified with lung cancer were at Stage-I: 41 of 54 (76%), including 12% with Stage-IA. The percentage of female patients (71%) and minority (51%) were also enriched in the cancer population. ANCOVA showed no significant effects for age, pack-years of smoking, gender or race on buccal *D* (p > 0.24).

**Table 2:** Demographic factor distribution for Sites 2. The Average value and standard deviation are reported.

Demographic factor	Control parameter	Cancer parameter	*p*-Value
	Site 2	Site 2	Site 2
Age	61 ± 11	67 ± 7	0.24
Pack-years	39 ± 34	41 ± 30	0.37
Gender (% female)	42%	71%	0.24
Race (% Caucasian)	49%	49%	0.99

Table 3 demonstrates that the AI-enhanced buccal csPWS distinguished all stages of lung cancer including the patients with Stage-I lung cancer from the control population with a robust AUC of 0.82 ± 0.09 (Se = 78%, Sp = 87%).

**Table 3:** Diagnostic performance of AI-enhanced csPWS microscopy for Stage-I, and all-Stage lung cancer in Site 2.

Lung cancer	AI-enhanced csPWS nanocytology (site 2)	
	AUC	(#Control, #case)
Stage-I	0.82 (± 0.11)	(43,40)
Stage-I and II	0.82 (± 0.04)	(43,44)
All-stages (I, II, III, and IV)	0.82 (± 0.09)	(43,54)

## Discussion

From a clinical perspective, early detection of lung cancer is critical to improving patient outcomes. Unfortunately, existing strategies, such as screening high-risk smokers using LDCT, have shortcomings. These include lack of uptake (estimated to be ~ 5%), false positives, etc^53^. This has provided the impetus for other approaches, especially blood-based biomarkers, including epigenetic (microRNA and methylation) and proteins secreted by tumor cells into the blood^54^. Although excellent for larger tumors, tumor-secreted biomarkers have drawbacks when applied to earlier lesions. This may be related to less secretion of tumor biomarkers in small early-stage (i.e., curable) tumors. Furthermore, especially for mutational or genomic biomarkers, the considerable tumor heterogeneity and later-stage mutations may not exist at a high level in small tumor lesions. The AI-enhanced technologies that evaluate hundreds of biomarkers and have some successes face challenges, as most of these biomarkers are not produced in sufficient quantities by early-stage, small tumors. Thus, the use of techniques relying on tumor secretions into the blood as the biomarker source tends to suffer from a drop in sensitivity for the small neoplastic lesions that diagnostically speaking, are of the greatest interest^12,55,56^. This is what led our team to seek alternate approaches to lung cancer screening.

There is growing evidence in support of lung field carcinogenesis with multiple genetic, epigenetic, metabolomic, and transcriptional alterations found throughout the aero-digestive mucosa in patients with lung cancer^15,19–23,57–60^. This suggests that patients who are genetically ‘programmed’ to have a pro-neoplastic response to a carcinogen like smoking (only ~ 10–20% of heavy smokers develop lung cancer). In particular, a variety of genetic/epigenetic changes in the buccal mucosa are concordant with those in lung cancer^16,17,23,58,61,62^.

Chromatin structure serves as the substrate which enables the genetic/epigenetic changes leading to neoplasia, and thus with the right technology, can be used as a predictor of cancer in histologically normal cells, even before the onset of tumor formation. Cytometry measurement^63^ and recent spectroscopic studies^18,19^ have indicated structural changes and field cancerization in the oral cavity of patients with lung cancer. Electron microscopy image analysis determined alterations in buccal chromatin packing at a length scale between 80 to 200 nm^51^, which is a profound and significant characteristic of field cancerization. Chromatin is organized into multiple packing domains, with an average diameter of 160 nm, demonstrating length scale invariant packing scaling behavior^44^. More specifically, the physical descriptor of packing scaling D is a statistical marker of chromatin conformation that is shown to be a predictor of transcriptional plasticity and correlated with the chance of survival among patients with cancer^26,30,33,44^. We hypothesized that the combination of field carcinogenesis as the biomarker source, and alterations in chromatin domain conformation as the biomarker type, can be utilized to develop a new lung cancer screening methodology that explores transcriptional plasticity-fostering chromatin domain alteration. As tumorigenesis occurs upon this fertile epigenetic field, its biomarkers should be indicative of lung cancer regardless of tumor size.

We developed csPWS nanocytology and utilized AI to optimize the combination of the biomarker source and type by employing field carcinogenesis. The buccal chromatin packing domain conformation (i.e., mapping chromatin packing scaling D across cell nuclei) in cells swabbed from the buccal epithelium was assessed using an AI-enhanced analysis of the intranuclear images of D. Convolutional and max-pooling layers from VGG16 trained on a vast ImageNet data set, successfully capturing distinctive features from the D-images. In particular, the early convolution layers capture low-level features of local patterns within the D image, while the intermediate and higher layers focus more on specific and complex structures within the global context. We determined a subset of features extracted from the final convolutional layers of blocks 2 through 5 containing valuable diagnostic information from D images and employed them for the detection of lung cancer. Our AI-enhanced biomarkers are developed with layers informed by mechanistically driven chromatin structural changes and have advantages over conventional biomarker discovery methods such as (1) single hypothesis-driven biomarkers and (2) the AI-enhanced “black-box” approaches. A single hypothesis-driven biomarker approach cannot fully capture the complexity of biological interactions while the “black-box” approach will fail to deliver an accurate diagnosis in a limited sample size due to the lack of mechanistic rationale. In this work, we bridged the two approaches while leveraging their strengths and mitigating their weaknesses for the detection of early-stage lung cancer. This is the reason our AI-enhanced csPWS microscopy exhibited significantly higher diagnostic performance (AUC = 0.92) for the detection of Stage-I lung cancer in comparison to the univariate assessment of buccal nuclear D (AUC = 0.75).

To test the rigor of our approach we specifically constructed this study from two distinct demographics and logistics for the discovery (Site 1) and validation datasets (Site 2). The discovery dataset was from a more affluent site and also required local transportation to the sample analysis site whereas the validation dataset was a safety net hospital with poverty and much larger portions of African-Americans, and Hispanic patients. Site 2 was located almost 2000 miles away from the analytical center which increases the chance of chromatin degradation during the out-of-state shipment. Importantly, the validation set was a reasonable approximation supporting the robustness of csPWS nanocytology. AI-enhanced csPWS nanocytology demonstrated high diagnostic performance for the detection of Stage-I lung cancer across the samples in Site 1 (AUC = 0.92, Se = 92%, Sp = 89%) and in Site 2 (AUC = 0.82, Se = 78%, Sp = 83%). This demonstrates a significant improvement over the current state-of-art technologies (Se < 25% for Stage-I lung cancer)^13,55^. In addition to high sensitivity to small lesions and Stage-I lung cancer, the diagnostic performance was independent of the tumor size and maintained its robust sensitivity for stages II, III, and IV. This high sensitivity to early-stage and late-stage lung cancer is possibly due to field carcinogenesis and our design of the biomarker source (buccal mucosa) and biomarker type (chromatin structure).

The performance of AI-enhanced csPWS nanocytology, especially for Stage-I disease, suggests that it may have a potential future role in the clinic. csPWS appears to outperform current blood tests for early-stage lung cancer screening, and while LDCT has showed a reasonable 20–25% reduction in lung cancer mortality, its effectiveness is limited by the fact that only about 5% of the eligible population undergoes LDCT screening^3^. Due to underreporting of pack-years, noncompliance with LDCT, and the rapidly increasing rates of lung cancer in non-smoking subjects (likely due to exposure to radon gas, air pollution, etc.) as well as quit-and second-hand smokers, a significant portion of lung cancer deaths now occurs among patients who would not even meet the criteria for LDCT screening^53^. In those undergoing LDCT screening, the benefits are often offset by harms from incidental findings (benign lesions that act as lung cancer mimics leading to unnecessary and costly testing, complications for invasive procedures and patient anxiety, etc.^53,64–66^). Indeed, for LDCT the number of patients needed to screen to prevent one death compared to the number needed to create harm was 1 in 130 versus 1 in 59, respectively^67^. Furthermore, overdiagnosis (detecting indolent and hence clinically insignificant disease) has been problematic, especially in the common “ground glass” lesions subjecting patients to surgical treatments that will not improve longevity. Risk stratification (enriching the LDCT population for lung cancer) has been increasingly popular. Demographic approaches such as the PLCO algorithm have been utilized^54^, however, there is increasing alarm at the incidence of lung cancer in those without traditional risk factors. Thus, buccal csPWS may be a compelling alternative approach.

Our study had some limitations. This clinical study used a case–control design with a limited number of patients. While the study shows that our AI-enhanced nanosensing technology can be a promising approach for the early detection of lung cancer, there is a need for further clinical data. Future work will build upon this study and involve a large-scale analysis to advance the AI model that was developed in the manuscript. Since most patients in this study have a history of smoking, future studies will validate our findings with a substantial non-smoker population. Additionally, there are some confounding variables that future work will further examine. As an example, the possible impact of shipment on chromatin degradation is unknown and needs to be determined in further studies. Similarly, the contribution of neoplastic signals from different layers of the buccal mucosa is not known, and the exact complex organization of packing domains is a subject of ongoing research. This suggests that further optimizations may lead to improved diagnostics.

In summary, our clinical data validate the need to optimize the combination of the biomarker source and type, as well as their choices (field carcinogenesis and chromatin alteration). Our data demonstrated that buccal csPWS was able to identify early-stage lung cancer with excellent accuracy, outperforming other purported minimally-invasive tests for screen-relevant neoplasia. The csPWS SOP is compatible with a simple at-home, self-administered collection of a buccal swab, or at the primary care physician or dentist’s office, which can then be shipped to a centralized laboratory for analysis. This strategy has the potential to significantly increase screening accessibility and uptake, and improve outcomes. The successful deployment of the field carcinogenesis/chromatin packing scaling D/AI-enhanced csPWS paradigm as a screening strategy for lung cancer in clinical practice may potentially enable the highly sensitive screening of a much larger portion of the asymptomatic at-risk or average-risk population, leading to increased detection of early-stage cancer, improved mortality, and fewer false positives and unnecessary procedures.

### Supplementary Information

Supplementary Figure S1.

## Data Availability

The raw datasets generated during and/or analyzed during the study are available from the corresponding author on reasonable request.
