# Supplementary figures and images for: Early detection of lung cancer using artificial intelligence-enhanced optical nanosensing of chromatin alterations in field carcinogenesis
Source: Sci Rep. 2023 Aug 22;13:13702. doi: 10.1038/s41598-023-40550-6 (PMC10444865; doi:10.1038/s41598-023-40550-6)

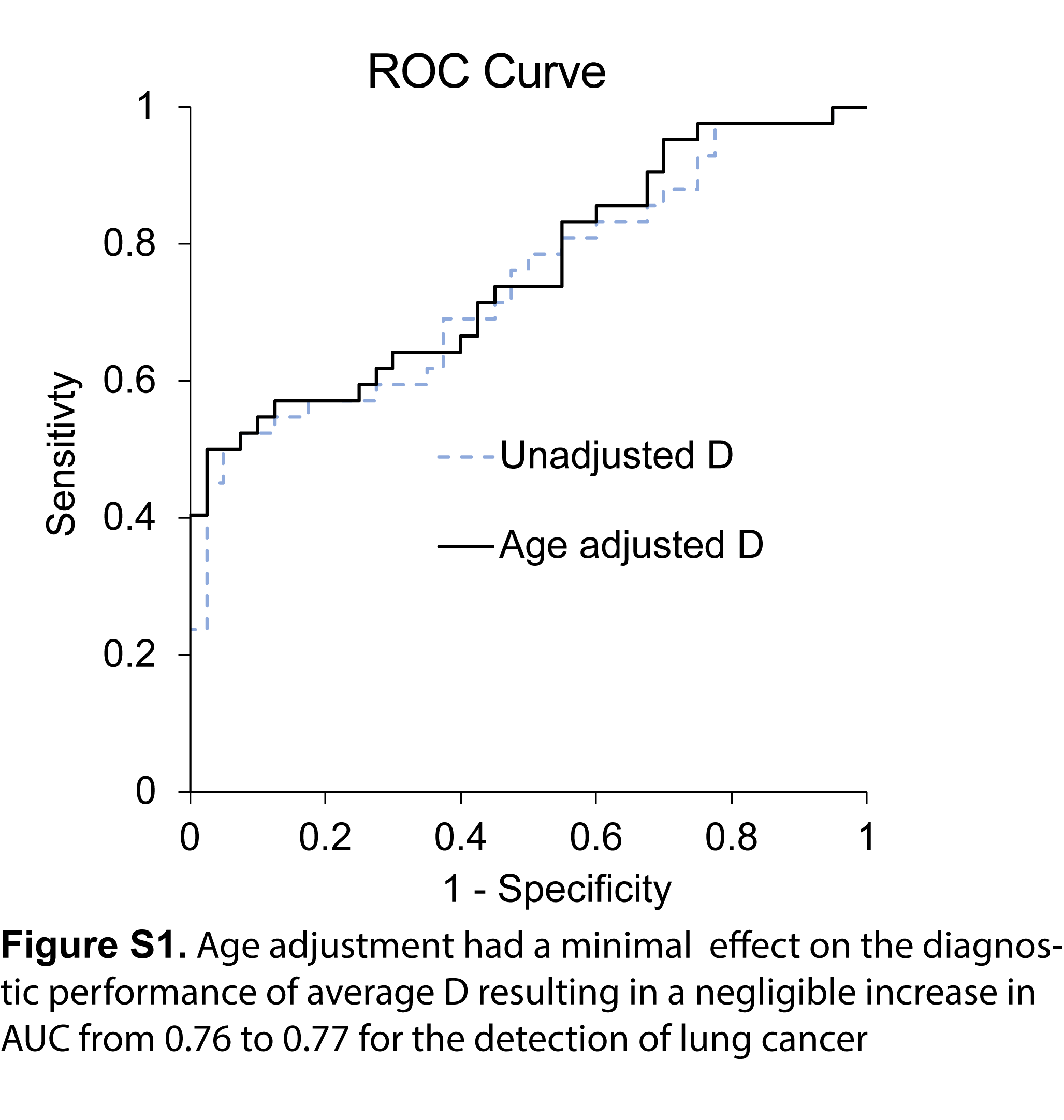

Supplement: Supplementary file 1 — Supplementary Figure S1. [file 41598_2023_40550_MOESM1_ESM.tif]
